# Analysis of the Clinical Significance and Safety of Interferon in the Treatment of Chronic Myeloproliferative Tumors

**DOI:** 10.1155/2022/6551868

**Published:** 2022-05-16

**Authors:** Fenglei Yin, Juan Yin, Weixing Xu, Shuchen Li, Wei Zhang, Juan Wang

**Affiliations:** ^1^Department of Hematology, Cangzhou Central Hospital, Cangzhou, China; ^2^Anesthesiology Deptartment and Operation Room, Cangzhou People's Hospital, Cangzhou, China

## Abstract

**Objective:**

To investigate the clinical significance and safety of interferon in the treatment of chronic myeloproliferative tumors (MPN).

**Methods:**

In this prospective study, a total of 120 patients with advanced chronic MPN admitted to our hospital between April 2016 and August 2020 were assessed for eligibility and recruited, including 62 patients with JAK2V617F mutation-positive ET (ET group) and 58 patients with JAK2V617F mutation-positive PV (PV group). 62 patients with JAK2V617F mutation-positive ET were assigned (1 : 1) to receive interferon-*α* (IFN-*α*) or hydroxyurea (HU). A similar subgrouping method for treatment of IFN-*α* and HU was introduced to patients with JAK2V617F mutation-positive PV. Outcome measures included efficacy and adverse reactions.

**Results:**

For patients with JAK2V617F mutation-positive ET and PV, there were no significant differences in the overall response rate between the groups treated with IFN-*α* or HU (*P* > 0.05); however, the patients treated with IFN-*α* had a significantly higher 5-year progression-free survival (PFS) than those treated with HU (*P* < 0.05). IFN-*α* was associated with a significantly lower incidence of disease progression, thrombotic events, splenomegaly, myelofibrosis, nausea, and vomiting and a higher incidence of hematological adverse reactions and flu-like symptoms versus HU (*P* < 0.05). After six months of treatment, the PV group had 12 cases of hematological response both in the IFN-*α* subgroup and the HU subgroup and fewer PV patients treated with IFN-*α* required phlebotomy versus those treated with HU (*P* < 0.05), in which 4 patients in the IFN-*α* subgroup had no hematological response and 6 patients in the HU subgroup had no hematological response. There was no significant difference in the number of cases with phlebotomy between the two subgroups of PV patients without hematological response (*P* > 0.05).

**Conclusion:**

The use of IFN in the treatment of JAK2V617F mutation-positive ET and PV patients yields a prominent clinical effect by prolonging PFS and avoiding phlebotomy for JAK2V617F mutation-positive PV patients.

## 1. Introduction

Myeloproliferative neoplasms (MPNs) are malignant clonal disorders of hematopoietic stem cells, clinically characterized by increased myeloid terminally differentiated cells [1]. It includes essential thrombocythemia (ET), polycythemia vera (PV), and primary myelofibrosis (PMF) [2]. Clinical related research has shown that about 75% of PV patients and about 45% to 55% of ET patients have mutations in the Janus kinase 2 (JAK2) gene (JAK2V617F) (the guanine at codon 617 in the JH2 region of the JAK2 gene was replaced by thymine, resulting in the replacement of valine by phenylalanine) [3]. Tian et al. [4] found that mutations in the gene JAK2 are associated with a higher blood cell load and a higher incidence of thrombotic events in clinical practice. The common clinical treatment of MPNs is based on the classification and risk stratification of the disease. Mostly, antiplatelet agents in combination with myelosuppressive drugs such as hydroxyurea (HU) or interferon (IFN) are used for the treatment of MPNs [5], but a clinical study has shown that long-term use of HU may induce second tumors [6]. Zhang et al. [7] stated that the use of IFN in the treatment of MPNs can effectively reduce the JAK2V617F gene allele load and result in a better hematologic response in patients. Complete remission can even be achieved in some patients without increasing the risk of mutagenesis [8]. At present, the application effect of interferon-*α* (IFN-*α*) in the treatment of MPN has been marginally explored in China. Although daily administration of IFN-*α* maintains higher blood concentrations versus alternate-day administration, the benefit from high blood concentrations is offset by the downregulation of interferon receptor expression over time, whereas the dosing interval in alternate-day administration facilitates the recovery of interferon receptor expressions. Interferon receptors and blood concentrations jointly determine clinical effects. Here, the present study was conducted to investigate the clinical efficacy and safety of IFN in the treatment of chronic MPN.

## 2. Materials and Methods

### 2.1. Study Flowchart

#### 2.1.1. Baseline Information

In this prospective study, a total of 120 patients with advanced chronic MPN admitted to our hospital between April 2016 and August 2020 were assessed for eligibility and recruited, including 62 patients with JAK2V617F mutation-positive ET (ET group) and 58 patients with JAK2V617F mutation-positive PV (PV group). 62 patients with JAK2V617F mutation-positive ET were assigned (1 : 1) to receive interferon-*α* (IFN-*α*) or hydroxyurea (HU). A similar subgrouping method for treatment of IFN-*α* and HU was introduced to patients with JAK2V617F mutation-positive PV. Due to the unique clinical characteristics, risk stratification, and treatment regimens of PMF patients, which are quite different from those of ET and PV patients, they were excluded in this study. All patients enrolled were regularly monitored for blood routine, liver and kidney function, and the progression of the disease. The patients' profiles are shown in Table 1. The study was approved by the Ethics Committee of the Cangzhou Central Hospital. The ethics certificate number of this study is 2015-11-12.

#### 2.1.2. Inclusion Criteria

Patients who met the WHO diagnostic criteria and were diagnosed with advanced MPN; patients with liver and kidney function that did not exceed 2 times the upper limit of normal at the initial treatment; patients without severe cardiac insufficiency and other complications; patients with no contraindications to the use of IFN or HU; and patients with good compliance to complete the entire treatment process were included.

### 2.2. Methods

1. Treatment with HU: the initial dose of HU for the patient was 10–15 mg/kg daily, and the dosage was adjusted according to the patient's treatment response. After the patient's blood profile returned to normal, a small dose of HU 0.3–0.45 g/d maintenance therapy plus aspirin 100 mg/d for adjuvant therapy were given.
2. IFN therapy: the patients received IFN-*α* 1b therapy. The therapy was performed daily or on alternate days through subcutaneous injection with an initial dose of 25–45 *μ*g. If patients had disease remission, the dosing frequency was changed to alternate days and was gradually reduced to 1–3 doses per week. The patients were additionally given aspirin 100 mg/d for adjuvant therapy. The treatment was given for more than 1 year. When the hematocrit of PV patients exceeded 50%, phlebotomy was adopted to reduce the patient's blood viscosity and the risk of thrombotic events.

### 2.3. Evaluation of Efficacy and Adverse Reactions

Efficacy evaluation criteria were referred to the prior literature [9]. Progression-free survival (PFS) refers to the duration from the date of enrollment to the first occurrence of disease progression (new thrombosis, bleeding events, progressive enlargement of the spleen, myelodysplastic syndrome (MDS), myelofibrosis (MF), aggravation of the original myelofibrosis reticulum staining, or acute myeloid leukemia (AML) caused by peripheral cytopenia), or from the date of enrollment to the time of death from any cause. Adverse reactions were evaluated with reference to the National Cancer Institute 3.0 standard, and the cases with adverse events were recorded.

### 2.4. Statistical Analysis

All data analyses were performed using SPSS 21.0. Measurement data are expressed as (mean ± SD) and analyzed using independent samples *t*-test. Count data are expressed as number of cases (rate) and analyzed using the chi-square test. Differences were considered statistically significant at *P* < 0.05.

## 3. Results

### 3.1. Clinical Efficacy

For patients with JAK2V617F mutation-positive ET and PV, there were no significant differences in the overall response rate between the groups treated with IFN-*α* or HU (*P* > 0.05); however, the patients treated with IFN-*α* had a significantly higher 5-year PFS than those treated with HU (*P* < 0.05) (Table 2).

### 3.2. Disease Progression and Adverse Reactions

IFN-*α* was associated with a significantly lower incidence of disease progression, thrombotic events, splenomegaly, myelofibrosis, nausea, and vomiting and a higher incidence of hematological adverse reactions and flu-like symptoms versus HU (*P* < 0.05) (Table 3).

### 3.3. Phlebotomy in Patients with PV

After six months of treatment, the PV group had 12 cases of hematological response both in the IFN-*α* subgroup and the HU subgroup and fewer PV patients treated with IFN-*α* required phlebotomy versus those treated with HU (*P* < 0.05), in which 4 patients in the IFN-*α* subgroup had no hematological response and 6 patients in the HU subgroup had no hematological response. There was no significant difference in the number of cases with phlebotomy between the two subgroups of PV patients without hematological response (*P* > 0.05) (Table 4).

## 4. Discussion

MPN-associated disease driver mutations include mutations in the janus kinase 2 (JAK2, chromosomal location 9p24) gene, myeloproliferative Leukemia Virus Oncogene (MPL, chromosomal location 1p 34) gene, and calreticulin (CALR, chromosomal location 19p13.2) gene. Different detection strategies are adopted for MPN. The basic strategy currently adopted is that all patients with clinically suspected PV are tested for the JAK2V617F mutation. If the JAK2V617F mutation is negative but there is still a high clinical suspicion of PV, testing for the JAK212 exon mutation or the LNK mutation is indicated. The JAK2V617F mutation is tested in patients with clinical suspicion of prothrombocytosis or primary myelofibrosis. If JAK2V617F is negative, further testing for MPL and CALR mutations is indicated. MPNs include chronic myelogenous leukemia (CML), PV, ET, and primary myelofibrosis.

IFN, as one of the cytokine family members, is often used clinically in the treatment of tumors and infectious and rheumatoid-related diseases such as lymphoma, leukemia, viral hepatitis, and rheumatoid arthritis [10]. INF is a cytokine with broad-spectrum antiviral, tumor growth inhibition, and immune regulation [11]. IFN-*α*, one of its isoforms, regulates the generation of the hematopoietic system by affecting human megakaryocyte precursors and immature pluripotent hematopoietic progenitor cells without increasing the risk of cellular mutagenesis, which may provide a viable treatment alternative for MPN patients [12]. The effect of the JAK2V617F mutant on the clinical phenotype of patients with MPN and the effectiveness of IFN-*α* against it have been confirmed by numerous clinical studies [13]. Li et al. [14] found that the gene load of JAK2V617F mutation in patients with MPN was closely related to the severity and duration of the disease. Zhang et al. [15] found that IFN-*α*specifically blocked the proliferation advantage of JAK2V617F mutant hematopoietic stem cells in mice, thereby preventing the development of MPN and even achieving eradication [16]. Clinical research has reported that some patients with MPN treated with IFN achieved complete hematologic and molecular biological remission [17]. However, the clinical efficacy of IFN on JAK2V617F mutation-positive patients has been marginally explored [18].

Adverse events such as fever, headache, and malaise associated with the administration of interferon (regular formulation) did not differ between daily and alternate-day administrations at the initial stage and were significantly less severe and less frequent in the weekly application group of the long-acting formulation. The adverse events such as fever, headache, chills, malaise, myalgia, and arthralgia of pegylated interferon *α* are less frequent and significantly milder, and patient compliance is good with once weekly dosing. However, its higher price compared to regular interferon has limited its widespread use in clinical practice. Given the costs and patient compliance, the clinically recommended regimen for IFN-*α* in myeloproliferative neoplasms is 3 million units (30 *μ*g) administered subcutaneously on alternate days.

The results of the present study showed that for patients with JAK2V617F mutation-positive ET and PV, there were no significant differences in the overall response rate between the groups treated with IFN-*α* or HU; however, the patients treated with IFN-*α* had a significantly higher 5-year progression-free survival (PFS) than those treated with HU. Moreover, IFN-*α* was associated with a significantly lower incidence of disease progression, thrombotic events, splenomegaly, myelofibrosis, nausea, and vomiting and a higher incidence of hematological adverse reactions and flu-like symptoms versus HU Also, it was found that after six months of treatment, the PV group had 12 cases of hematological response both in the IFN-*α* subgroup and the HU subgroup and fewer PV patients treated with IFN-*α* required phlebotomy versus those treated with HU, in which 4 patients in the IFN-*α* subgroup had no hematological response and 6 patients in the HU subgroup had no hematological response. There was no significant difference in the number of cases with phlebotomy between the two subgroups of PV patients without hematological response. All these suggest that IFN treatment of patients with chronic MPN can effectively improve the long-term PFS of patients [19]. Moreover, the incidences of thrombotic events, splenomegaly, and myelofibrosis in patients treated with IFN were significantly lower than in those treated with HU [20]. The adverse reactions of patients treated with IFN are mainly flu-like symptoms, which are relieved after discontinuation of treatment or application of symptomatic drug treatment that is tolerated by most patients. For PV patients, the use of IFN therapy can effectively avoid phlebotomy [21].

To sum up, the use of IFN in the treatment of JAK2V617F mutation-positive ET and PV patients yields a prominent clinical effect by prolonging PFS and avoiding phlebotomy for JAK2V617F mutation-positive PV patients.

## Figures and Tables

**Table 1:** Baseline data (*n* (%)).

	ET (*n* = 62)		t/*x*^2^	*P*
	INF-*α* (*n* = 32)	HU (*n* = 30)		
Gender (*n*)			0.0	0.986
Male	17	16		
Female	15	14		
Median age (years)	53.47 ± 5.61	53.60 ± 5.73	−0.091	0.928
WBC (×10^9^/L)	13.72 ± 3.64	13.68 ± 3.59	0.035	0.972
Hb (g/L)	135.27 ± 19.42	135.37 ± 18.94	−0.021	0.983
PLT (×10^9^/L)	1420.81 ± 411.32	1433.72 ± 412.26	−0.123	0.902
Risk stratification (*n*)			0.001	0.974
Low risk with extreme thrombocytosis	14	13		
High risk with extreme thrombocytosis	18	17		

	PV (*n* = 58)		t/*x*^2^	*P*
	INF-*α* (*n* = 29)	HU (*n* = 29)		

Gender (*n*)			0.069	0.792
Male	16	15		
Female	13	14		
Median age (years)	54.31 ± 5.61	54.24 ± 5.81	0.046	0.963
WBC (×10^9^/L)	15.12 ± 4.53	15.28 ± 4.62	−0.126	0.900
Hb (g/L)	201.33 ± 20.11	202.22 ± 20.21	−0.168	0.867
PLT (×10^9^/L)	441.12 ± 200.32	442.51 ± 199.36	−0.026	0.979
Risk stratification (*n* (%))			0.07	0.791
Low risk with extreme thrombocytosis	16	17		
High risk with extreme thrombocytosis	13	12		

**Table 2:** Comparison of effective rates of ET and PV patients (*n* (%)).

Groups	OS	Cr	Pr	5-year PFS rate
ET (*n* = 62)				
INF-*α* (*n* = 32)	29 (90%)	20 (63%)	8 (25%)	27 (84%)
HU (*n* = 30)	26 (87%)	17 (57%)	9 (30%)	16 (53%)
*x* ^2^	0.242	0.219	0.195	7.02
*P*	0.623	0.64	0.659	0.008

Groups	OS	Cr	Pr	5-year PFS rate

PV (*n* = 58)				
INF-*α* (*n* = 29)	25 (86%)	20 (69%)	5 (17%)	25 (86%)
HU (*n* = 29)	24 (83%)	16 (55%)	8 (28%)	15 (52%)
*x* ^2^	0.132	1.172	0.892	8.056
*P*	0.717	0.279	0.345	0.005

**Table 3:** Comparison of disease progression and adverse reactions in patients (*n* (%)).

	INF-*α* (*n* = 61)	HU (*n* = 59)	*x* ^2^	*P*
Disease progression	14 (23%)	27 (46%)	6.939	0.008
Thrombotic event	11 (18%)	23 (39%)	6.483	0.011
Splenomegaly	9 (15%)	21 (36%)	6.946	0.008
Myelofibrosis	8 (13%)	17 (29%)	4.482	0.034
Adverse reaction				
Hematologic adverse reactions			7.592	0.006
Slight	7 (11%)	16 (27%)		
Serious	0 (0%)	3 (5%)		
Flu-like symptoms	42 (69%)	0 (0%)	62.497	<0.001
Nausea and vomiting	0 (0%)	6 (10%)	6.53	0.011
Allergy	4 (7%)	3 (5%)	0.118	0.731
Hepatic dysfunction	1 (2%)	4 (7%)	1.985	0.159

**Table 4:** Comparison of bloodletting rates with different treatment methods in PV patients (*n* (%)).

Phlebotomy rate	IFN-*α* (*n* = 12)	HU (*n* = 12)	x^2^	*P*
Overall hematologic response	1 (8%)	7 (58%)	6.75	0.009

No response	3 (75%)	4 (67%)	0.202	0.653

## Data Availability

The datasets used during the present study are available from the corresponding author upon reasonable request.
